# Li BO—Pioneer of Ecology in China

**DOI:** 10.1007/s13238-016-0286-1

**Published:** 2016-07-05

**Authors:** Zhicheng Gao

**Affiliations:** Inner Mongolia University, Hohhot, 010000 China

**Figure 1:**
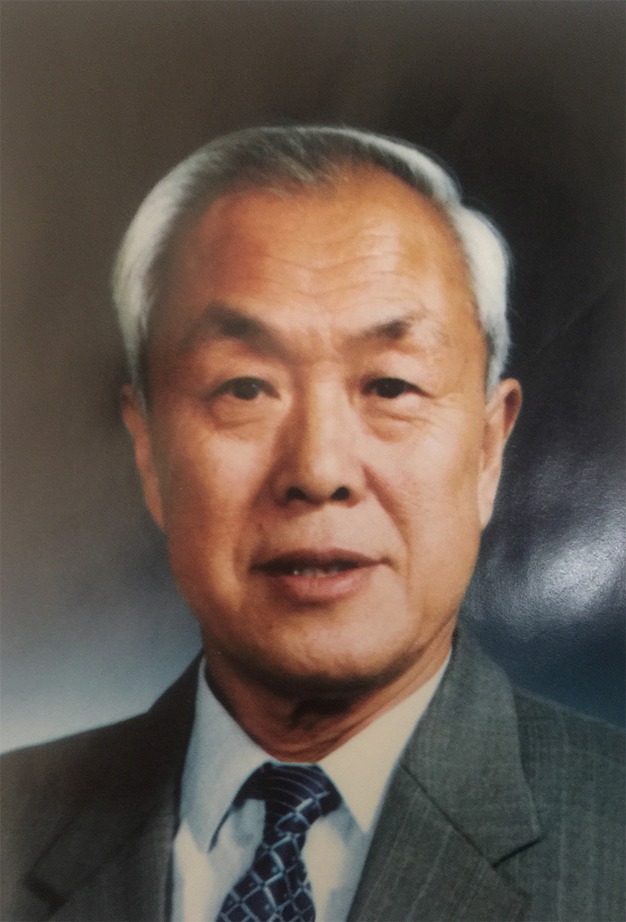
Li Bo (1929–1998)

“Li Bo was the founder of Plant Ecology in China, the successor of Li Jitong and the pioneer of botany and phytophysiology of China.” (Wu Zhengyi, 1980) Li Bo was a brilliant ecologist and botanist. He established the science of Ecology in China in the early 1970s. He conducted a comprehensive and systematic study on vegetation in arid and semi-arid regions using his knowledge of Ecology and combining basic research with applied study as well as traditional and modern research methods. By involving global change, sustainable development and biodiversity conservation, Li Bo linked Ecology in China to hot issues worldwide. His creative idea of applying remote sensing (RS) and Geographic Information System (GIS) to pasture resource investigation in 1980s promoted the conservation of Grassland Ecology to a new level (Fig. 1).

Li Bo () was born in Shandong Province on April 15th, 1929. After graduating from Beijing Agricultural University in 1953, Li Bo worked in Peking University where he met his supervisor Li Jitong, who was noted for his studies of plant biology and for devoting himself to the development of Ecology. (Li B, 1979) Li Bo started his research with Li Jitong in 1955. At that time, he knew almost nothing about how to investigate the vegetation on Xishan mountain of Beijing. He spent two years learning basic knowledge and English which laid a solid foundation for his following research career. Subsequently, he went to Hulun Buir grassland in Inner Mongolia to explore how the vegetation grew in this region. In that period, advanced research tools and transportation such as aerial photographs and vehicles were not available. The only means of transport was horse-drawn carriage. In such circumstances, with the guidance of Li Jitong, Li Bo began to take a strong interest in Grassland Ecology. From the first step on that fascinating area, although he wasn’t aware of it, Li Bo had become closely tied to grassland.

As an ambitious youth, Li Bo tried his best to support the construction of border areas around the grasslands two years later after his graduation. His wife Mrs. Jiang Peihua, who sacrificed her career, strongly supported Li Bo’s decision by following him wherever he went. The couple soon settled in Inner Mongolia. In 1958, Li Bo joined the team of Chinese Academy of Sciences combating desertification and started an integrated survey of desert ecology. In May 1959, Li Bo and his team arrived at Badain Jaran Desert, where even camels struggled to survive as the dune surface temperature rose close to 70°C. After more than 20 tough days, they obtained the primary data of flora, vegetation, hydrology and landforms that revealed the true features of the desert for the first time. Li Bo published several research papers illustrating the patterns of vegetation types and desert areas of Inner Mongolia. Furthermore, Li Bo put forward a zonal division and partition scheme in the early 1960s.

In the compiling of *Vegetation in China* in 1970s, Li Bo was the deputy leader of compilation group which described the vegetation conditions in arid and semi-arid regions of China (Li B, 1990a). Li Bo summarized the basic laws of grassland vegetation which were essential to the book and created a brand new classification system of vegetation with Chinese characters.

Li Bo was invited to pay an academic visit to University of Idaho in October 1981. While there he observed and studied prairies in 21 States of the USA, and perceived a huge academic gap between China and America. When he came back to China, he presided at the program of *Present Development in the Application of Remote Sensing to Grassland Resources Survey in China* which updated the evaluation and map-making techniques in 1983 (Li B, 1990b). This program involved almost one hundred specialists and nine universities in the ecological field.

**Figure 2:**
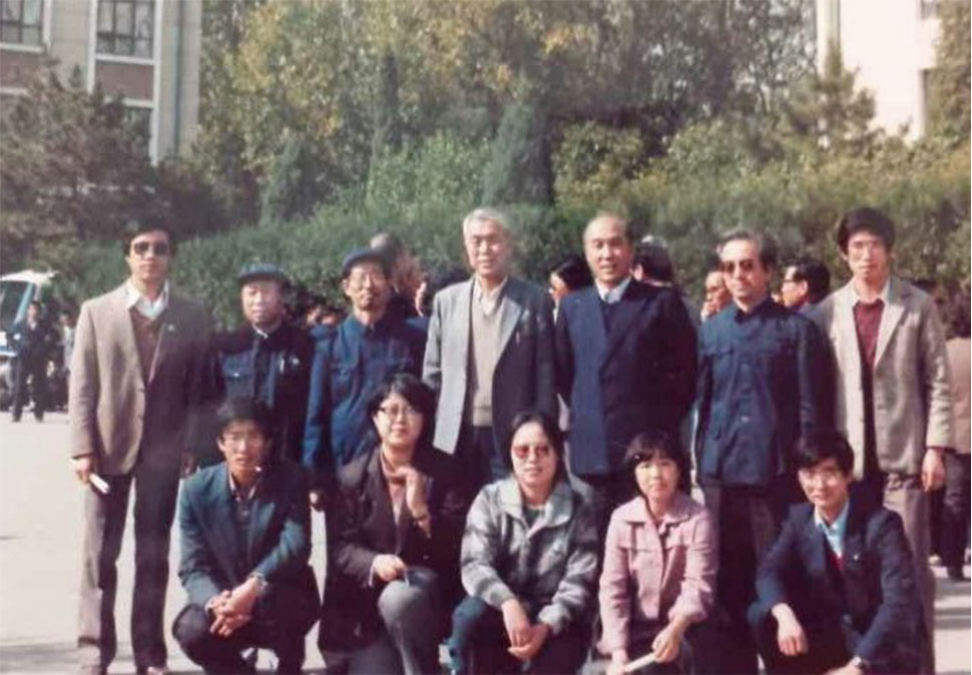
Li Bo (The 4th from left, line 2) in the international Association for Ecology

As the major organizer, Li Bo presided over 3 international academic conferences in Hohhot: the International Grassland Vegetation Congress, the International Session on Grassland Resources and the International Conference on Grassland Management in Mongolia. Besides these conferences, the International Association for Ecology led by Inner Mongolia University was highly influential in 1980s (Fig. 2). At that time, the Ecology department of Inner Mongolia University had gained a significant reputation in China and even in the world. The paper “The Sustainable Development of Deteriorated Grassland” written by Li Bo expounded the measures of grassland restoration was widely recognized and appreciated internationally.

**Figure 3:**
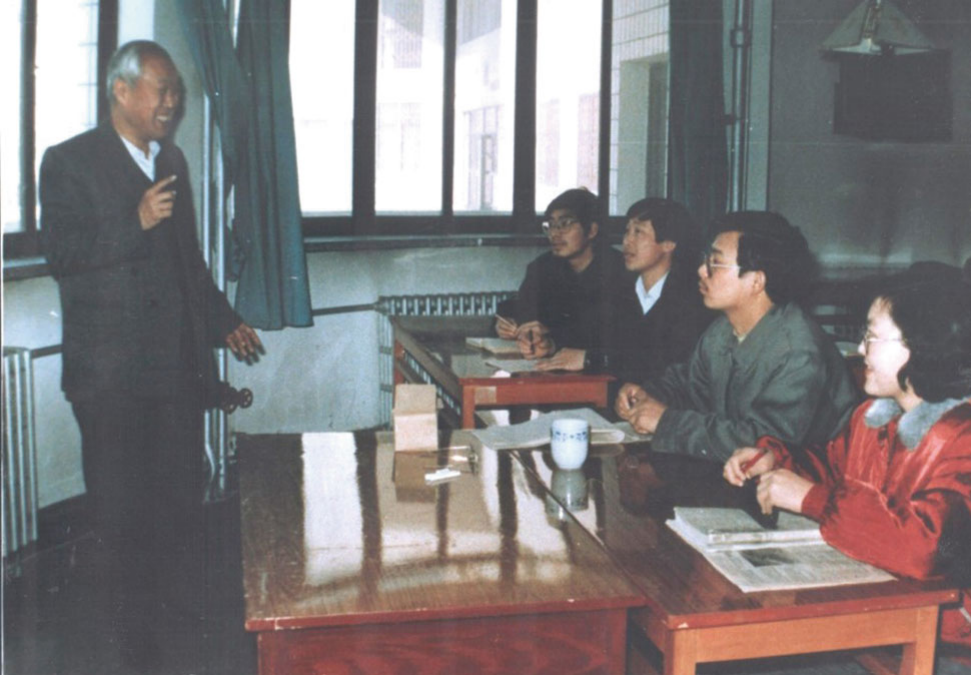
Li Bo was giving a brisk lecture to doctors of Ecology

Li Bo was the founder of the first specialty of Ecology in China. He had been teaching in Inner Mongolia University for more than 45 years. To fill the 20 years gap between Ecology in China and international level, Li Bo made painstaking efforts to find how to teach students and help them develop better scientific studies. He made new plans and targets of inter-disciplinary teaching methods, including English, Mathematics, Biostatistics and even computer application. Field trips and labs were also enhanced under his supervision and many textbooks were written or translated by him. In order to be more efficient in teaching, Li Bo invited many experts to present lectures about teaching methods of Ecology. Due to his effective teaching model, the first graduates were well recognized in their work of modern Ecology. Li Bo set up the first Master Degree program in 1978 and the first Ecology PhD program of China in 1990. His contributions to Inner Mongolia University helped the university become one of the most important talent training bases of Ecology in China (Fig. 3).

“I was cultivated by my country. So I will devote myself into Ecology, constructing my country and developing grassland science for the rest of my life.” Li Bo said when he was selected as the academician of Chinese Academy of Sciences in 1993. Li Bo, the founder of Ecology in China, enjoyed teaching and researching Ecology all his life. The textbook of General Ecology written by him is still used by college students today.
